# Increased risk of malignancy in patients with systemic lupus erythematosus: population-based cohort study in Korea

**DOI:** 10.1186/s13075-021-02648-y

**Published:** 2021-10-27

**Authors:** Jung-Yong Han, Hyoungyoung Kim, Sun-Young Jung, Eun Jin Jang, Soo-Kyung Cho, Yoon-Kyoung Sung

**Affiliations:** 1 Department of Rheumatology, Hanyang University Hospital for Rheumatic Diseases, Seoul, South Korea; 2 College of Pharmacy, Chung-Ang University, Seoul, South Korea; 3 Department of Information Statistics, Andong National University, Andong, South Korea

**Keywords:** Systemic lupus erythematosus, Malignancy, Incidence rate, Standardised incidence ratio

## Abstract

**Background:**

This study aimed to evaluate the crude incidence rates and relative risk of malignancy in Korean patients with SLE.

**Methods:**

We conducted a retrospective nationwide cohort study using databases from the National Health Insurance Service in Korea. All prevalent SLE patients aged over 19 were identified from January 2012 to December 2014 and observed until the diagnosis of malignancy, death, or end of the study, December 2015. The crude incidence rates (IRs) and standardised incidence ratios (SIRs) of overall and site-specific malignancies in SLE patients were estimated.

**Results:**

We identified 17,854 SLE patients and during the observation period (60,511 person-years [PYs]), 768 solid malignancies (126.9/10,000 PYs) and 68 haematologic malignancies (11.2/10,000 PYs) occurred in SLE patients. In SLE patients, breast and reproductive system and thyroid cancers occurred predominantly, followed by liver and colon cancers. The SIRs of overall, solid, and haematologic malignancies of SLE patients compared to the general population were 1.8 (95% confidence interval [CI] 1.6–1.9), 1.7 (95% CI 1.5–1.8), and 5.9 (95% CI 4.8–7.3), respectively. In solid malignancies, head and neck (2.7, 95% CI 1.1–4.2), bladder (2.4, 95% CI 1.1–3.8), liver (1.9, 95% CI 1.4–2.3), pancreas (1.9, 95% CI 1.3–2.6), lung (1.8, 95% CI 1.2–2.4), colon (1.7, 95% CI 1.3–2.2), thyroid (1.6, 95% CI 1.3–1.8) and breast and reproductive system (1.5, 95% CI 1.2–1.7) cancers are at increased risk in SLE patients.

**Conclusion:**

An increased risk of haematologic and solid malignancies was observed in Korean patients with SLE compared to the general population.

## Background

Systemic lupus erythematosus (SLE) is a relatively common systemic autoimmune rheumatic disease with a wide range of clinical presentations resulting from the involvement of multiple organ systems [1–5]. It commonly affects young and middle-aged people and is characterised by a 9:1 female to male ratio of disease incidence [6]. Although survival in SLE has improved during the past 30 years, its mortality remains high relative to the general population, and significant sex, racial, and regional disparities persist [7, 8].

The risk of malignancy in SLE has become an increasing concern because cumulative immunosuppressant drugs may be associated with increased cancer risk [9]. There is increasing evidence to suggest that patients with SLE have a higher overall risk of malignancy [10]. A recent multisite international cohort study reported an association between SLE and cancer (standardised incidence ratio, SIR, 1.14, 95% CI 1.05–1.23), highlighting the risk for non-Hodgkin lymphoma (NHL) and leukaemia, but also demonstrating an increased risk of vulvar, lung, thyroid and possibly liver cancers. On the other hand, it was reported that patients with SLE appear to have a decreased risk of breast, endometrial and ovarian cancers [11]. However, another comprehensive meta-analysis established epidemiologic evidence to support an association between SLE and increased risk for 16 cancers, except for prostate cancer and cutaneous melanoma [12].

Evidence of relationships between cancer and SLE has been provided in some studies of Korean populations [13, 14]. SLE patients were found to have more risk of malignancy, such as cervical cancer, lymphoma and bladder cancer. However, the subjects in these previous studies were patients selected from specific centres, not a population-based nationwide level.

We therefore evaluated the incidence rates and relative risk of malignancy in all Korean patients with SLE compared to the general population in this study.

## Methods

### Data sources

#### National claims database of Korea for SLE patients

The Health Insurance Review and Assessment Service (HIRA) database, which includes the Korean population of around 50 million individuals, was the primary data source for this study. The HIRA database contains information about individual beneficiaries, and healthcare service information such as diagnosis, procedure, prescription and diagnostic tests from in- and outpatient care.

#### National Health Insurance Service-National Sample Cohort Database (NHIS-NSC) for the general population

NHIS has developed a National Sample Cohort (NSC) database using stratified random sample for age, gender, participants’ eligibility stats, region and income level. A representative sample cohort of 1,025,340 participants, comprising 2% of the total Korean population, was included in this study [15].

### Study populations

#### Patients with SLE

We identified all of the Korean patients with SLE who were registered in the rare intractable disease (RID) registration programme in Korea. All patients in the programme had to have received a specific RID code based on the 1997 Update of the 1982 American College of Rheumatology Revised Criteria for Classification of Systemic Lupus Erythematosus [16]. Fulfilment of the criteria is reviewed strictly by the healthcare institution, because patients registered in this programme were supported financially by government aid.

**Fig. 1:**
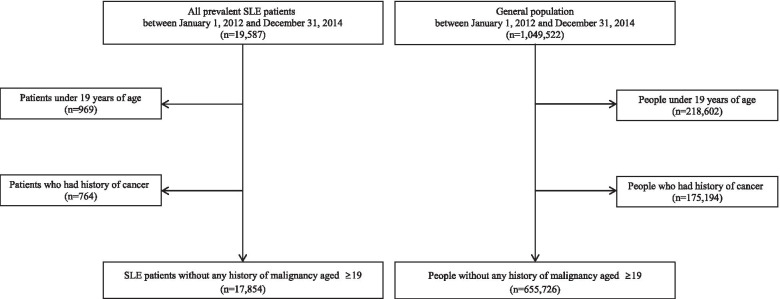
Selection flow of study population

Using this HIRA database, patients with both an ICD-10 code (M32.0) and RID code (V136) were defined as SLE patients. All the prevalent SLE patients aged ≥ 19 years registered in the RID programme between 2012 and 2014 were included in our study. Patients who had other connective tissue diseases such as rheumatoid arthritis or Sjögren's syndrome were excluded. To identify incident malignancy cases, we excluded patients who had a history of any malignancy in the 12 months before the index date (Fig. 1).

#### General population as a control group

As a comparator, we recruited individuals in the general population who had any medical claims from the NHIS-NSC database between 2012 and 2014 with matching the calendar years. After that, we excluded the people aged under 19 and those who had claimed any malignancy 12 months before the index date.

### Definition of incident malignancy

The occurrence of malignancy was regarded as the first claims under ICD-10 codes, such as C00 to C97, including both solid and haematologic malignancies according to the type or site of malignancy. Among solid malignancies, breast, cervix, corpus ovary, prostate and testis malignancies were grouped as breast and reproductive system malignancies and specific codes for each malignancy were masked. We set the date of the first claims of each malignancy as the date that the diagnosis of the malignancy was made. In cases where several malignancies occurred at different time points, we regarded every malignancy as independent incident cases during the observations [17].

The index date was defined as the date of the first claim for diagnosis with SLE combined with RID code or first claim of the control group for the observation period from 2012 to 2014. The previous one year before the index date was defined as the history period. Patients were followed from the index date to the date of malignancy diagnosis, death, or the end of 2015 if any malignancy was not diagnosed. If the patients were diagnosed with multiple types of malignancies, the follow-up period was established with the date of the last diagnosis of any type of malignancy.

### Statistical analysis

We calculated the crude incidence rate (IR) of malignancies per 10,000 person-year (PYs) in patients with SLE. Crude IR was calculated by dividing the incident cases of malignancies by the total observational periods of patients with a 95% confidence interval (CI). The crude IR of overall and each malignancy according to gender was presented in the descriptive analysis.

To estimate the risk of malignancy in patients with SLE compared to the general population, we calculated standardised incidence ratios (SIRs) by dividing the observed number of malignancies of SLE patients by the expected number of malignancies calculated from the accumulated PYs and age, gender, and calendar period-specific malignancy IRs of the general population. The SIR was described with 95% CI. All statistical analyses were performed using SAS 9.2 (SAS Institute Cary, NC)

## Results

### Baseline characteristics

Among the 19,587 SLE patients extracted from the NHIS database, 969 patients under 19 years of age and 764 patients with a previous history of cancer were excluded. We finally identified 17,854 SLE patients and most of these were female (women, *n*=16,143 and men, *n*=1,711, 90.4%).

Among 1,049,522 people from the general population in the NHIS-NSC database, we excluded 218,602 people under 19 years of age and 175,194 patients with a previous history of cancer. Finally, we included 649,010 people from the general population (women, *n*=306,649 and men=342,361) in this analysis as a control group.

Baseline characteristics for SLE patients and the general population are shown in Table 1. Most patients with SLE were women (90.4%) and younger than the general population. Compared to the general population, comorbidities were more common in patients with SLE (Table 1).

**Table 1:** Baseline characteristics of study population

Variables	SLE patients (*n*=17,854)	General population (*n*=649,010)
Age, years	42.3 ± 13.2	44.2 ± 16.1
Sex, female	16,143 (90.4)	306,649 (47.2)
Duration of observation, years	3.4 ± 0.9	3.7 ± 0.6
Payer type		
National health insurance	16,752 (93.8)	630,446 (71.1)
Medical aid	1102 (6.2)	18,564 (2.9)
Type of institution		
Tertiary hospital	12,362 (69.2)	15,059 (2.3)
General hospital	3837 (21.5)	30,927 (4.8)
Community hospital	146 (0.8)	36,687 (5.6)
Clinic	1377 (7.7)	239,369 (36.9)
Other	132 (0.7)	326,968 (50.4)
Number of comorbidities	1.2 ± 0.6	0.1 ± 0.3
Comorbidity		
Myocardial infarction	21 (0.1)	468 (0.07)
Congestive heart failure	162 (0.9)	1437 (0.2)
Peripheral vascular disease	512 (2.9)	4280 (0.7)
Cerebrovascular disease	253 (1.4)	3733 (0.6)
Dementia	38 (0.2)	132 (0.02)
Chronic pulmonary disease	492 (2.8)	12,724 (2.0)
Peptic ulcer disease	1084 (6.1)	10,119 (1.6)
Mild liver disease	920 (5.2)	7558 (1.2)
Diabetes mellitus	601 (3.4)	13,323 (2.1)
Hemiplegia or paraplegia	51 (0.3)	685 (0.1)
Renal disease	474 (2.7)	1,180 (0.2)
Moderate or severe liver disease	11 (0.1)	157 (0.02)
Charlson Comorbidity Index score	0.30 ± 0.69	0.10 ± 0.40

Numerical quantitative data were presented by “mean ± SD” and categorical data were presented by “frequency (%)”

### Crude IR of malignancy in patients with SLE and the general population

During the observation period (60,511 PYs), 836 overall malignancies (IR 138.16/10,000 PYs, 95% CI 128.79-147.52) occurred in SLE patients, compared with 22,735 events (IR 95.71/10,000 PYs, 95% CI 94.46-96.95) in the general population (2,375,523 PYs). In total, 768 incidents of solid malignancy (IR 126.9/10,000 PYs, 95% CI 117.94–135.90) and 68 incidents of haematologic malignancy (IR 11.24/10,000 PYs, 95% CI 8.57–13.91) occurred in SLE patients. For the number of site-specific malignancy cases diagnosed in SLE patients, breast and reproductive system (crude IR 28.9, 95% CI 24.6–33.2) has the highest IR, followed by thyroid (crude IR 25.4, 95% CI 21.4–29.5), liver (crude IR 10.4, 95% CI 7.8–12.9) and colon (crude IR 10.1, 95% CI 7.6–12.6) (Table 2).

**Table 2:** The crude incidence rate of malignancies in patients with SLE

Type of malignancy	Total (*n*=17,854)		Female (*n*=16,143)		Male (*n*=1711)	
	No. of cases	Incidence per 10,000 PYs (95% CI)	No. of cases	Incidence per 10,000 PYs (95% CI)	No. of cases	Incidence per 10,000 PYs (95% CI)
Overall malignancies	836	138.16 (128.79, 147.52)	738	134.45 (124.75, 144.15)	98	174.40 (139.87, 208.93)
Solid malignancy	768	126.92 (117.94, 135.90)	686	124.97 (115.62, 134.33)	82	145.93 (114.34, 177.51)
Breast and reproductive system	175	28.92 (24.64, 33.21)	164	29.88 (25.30, 34.45)	11	19.58 (8.01, 31.14)
Thyroid	154	25.45 (21.43, 29.47)	148	26.96 (22.62, 31.31)	6	10.68 (2.13, 19.22)
Liver	63	10.41 (7.84, 12.98)	55	10.02 (7.37, 12.67)	8	14.24 (4.37, 24.10)
Colon	61	10.08 (7.55, 12.61)	51	9.29 (6.74, 11.84)	10	17.80 (6.77, 28.83)
Lung	34	5.62 (3.73, 7.51)	25	4.55 (2.77, 6.34)	9	16.02 (5.55, 26.48)
Pancreas	32	5.29 (3.46, 7.12)	29	5.28 (3.36, 7.21)	3	5.34 (0, 11.38)
Stomach	31	5.12 (3.32, 6.93)	27	4.92 (3.06, 6.77)	4	7.12 (0.14, 14.09)
Bladder	12	1.98 (0.86, 3.11)	10	1.82 (0.69, 2.95)	2	3.56 (0, 8.49)
Head and neck	11	1.82 (0.74, 2.89)	9	1.64 (0.57, 2.71)	2	3.56 (0, 8.49)
Kidney	10	1.65 (0.63, 2.68)	5	0.91 (0.11, 1.71)	5	8.90 (1.10, 16.70)
Biliary tract	7	1.16 (0.30, 2.01)	4	0.73 (0.01, 1.44)	3	5.34 (0, 11.38)
Brain and central nervous system	5	0.83 (0.10, 1.55)	5	0.91 (0.11, 1.71)	0	NC
Larynx	2	0.33 (0, 0.79)	2	0.36 (0, 0.87)	0	NC
Haematology malignancy	68	11.24 (8.57, 13.91)	52	9.47 (6.90, 12.05)	16	28.47 (14.52, 42.43)
Non-Hodgkin lymphoma	36	5.95 (4.01, 7.89)	27	4.92 (3.06, 6.77)	9	16.02 (5.55, 26.48)
Multiple myeloma	14	2.31 (1.10, 3.53)	13	2.37 (1.08, 3.66)	1	1.78 (0, 5.27)
Leukaemia	12	1.98 (0.86, 3.11)	8	1.46 (0.45, 2.47)	4	7.12 (0.14, 14.09)
Hodgkin lymphoma	3	0.50 (0, 1.06)	3	0.55 (0, 1.16)	0	NC

*PY*, person-year; *CI*, confidence interval; *NC*, not calculated

### Increased risk of malignancy in SLE patients compared to the general population

Our study found SLE to be associated with increased risk of both solid (SIR 1.65, 95% CI 1.53–1.77) and haematologic (SIR 5.87, 95% CI 4.48–7.27) malignancies. For the risk of solid malignancy in SLE patients, head and neck cancer (SIR 2.7, 95% CI 1.1–4.2) had the highest risk, followed by bladder (SIR 2.4, 95% CI 1.1–3.8), liver (SIR 1.9, 95% CI 1.4–2.3), pancreas (SIR 1.9, 95% CI 1.3–2.6), lung (SIR 1.8, 95% CI 1.2–2.4), colon (SIR 1.7, 95% CI 1.3–2.2), thyroid (SIR 1.6, 95% CI 1.3–1.8) and breast or reproductive system (SIR 1.5, 95% CI 1.2–1.7) cancers. The risk of solid malignancy overall increased more in SLE patients than in the general population except for brain and central nervous system cancer (SIR 0.91, 95% CI 0.11–1.72).

**Fig. 2:**
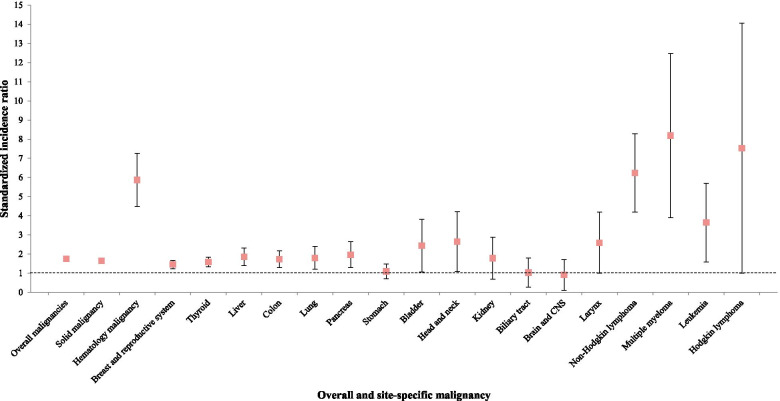
Increased risk of malignancy in Korean patients with SLE compared to the general population

In regards to haematologic malignancies, SLE patients had a relatively high risk for multiple myeloma (SIR 8.19, 95% CI 3.90–12.48), followed by NHL, (SIR 6.24, 95% CI 4.20–8.27) and leukaemia (SIR 3.64, 95% CI 1.58–5.70). However, the SIR of Hodgkin lymphoma (SIR 7.53, 95% CI 1.00–16.05) was not significantly high in Korean patients with SLE (Fig. 2).

### Sex differences in the risk of malignancy

There are some differences in the risk of malignancy between female and male SLE patients. Male SLE patients experienced higher crude IR of solid and haematologic malignancies than female SLE patients (Table 2). Female SLE patients had the highest crude IR of breast and reproductive system (crude IR 29.88/10,000PYs, 95% CI 25.30–34.45) cancer, followed by thyroid (crude IR 26.96/10,000PYs, 95% CI 22.62–31.31) and liver (crude 10.32/10,000PYs, 95% CI 7.37–12.67) cancer. Otherwise, male SLE patients had the highest crude IR of reproductive system cancer including prostate cancer (crude IR 19.58/10,000PYs, 95% CI 8.01–31.14), followed by colon (crude IR 17.80/10,000PYs, 95% CI 6.77–28.83) and lung (crude IR 16.02/10,000PYs, 95% CI 5.55–26.48) cancer and NHL (crude IR 16.02/10,000PYs, 95% CI 5.55–26.48).

For the SIR of malignancy in female SLE patients compared to the general population, multiple myeloma (SIR 8.95, 95% CI 4.08–13.82) was highest, followed by NHL (SIR 5.33, 95% CI 3.32–7.34) and leukaemia (SIR 3.64, 95% CI 1.58–5.70). In solid malignancy, bladder cancer (SIR 2.74, 95% CI 1.04–4.44) had the highest incremental risk in female patients with SLE. Other solid malignancies such as thyroid, breast and reproductive system, liver, colon, lung and pancreas cancers also showed a higher risk in female patients with SLE than in the general population (Table 3).

In male SLE patients, the SIR of NHL (SIR 12.71, 95% CI 4.41–21.02) was increased compared to the general population, but the risk of other haematologic malignancies such as leukaemia (SIR 7.80, 95% CI 0.16–15.45) and multiple myeloma (SIR 3.90, 95% CI 0–11.53) did not significantly increase. Although the risk of overall solid malignancies in male patients with SLE was increased (SIR 1.78, 95% CI 1.39–2.16), the risk of site-specific solid cancers was not increased compared to the general population (Table 3).

**Table 3:** Sex differences in SIRs of malignancy in patients with SLE compared to general populations

Type of malignancy	Total (*n*=17,854)	Female (*n*=16,143)	Male (*n*=1711)
Overall malignancies	**1.75 (1.63, 1.87)**	**1.72 (1.60, 1.84)**	**2.05 (1.65, 2.46)**
Solid malignancy	**1.65 (1.53, 1.77)**	**1.64 (1.51, 1.76)**	**1.78 (1.39, 2.16)**
Breast and reproductive system	1.45 (1.23, 1.66)	1.43 (1.21, 1.65)	1.76 (0.72, 2.81)
Thyroid	1.58 (1.33, 1.82)	1.55 (1.30, 1.80)	2.56 (0.51, 4.61)
Liver	1.86 (1.40, 2.32)	1.98 (1.46, 2.50)	1.32 (0.40, 2.23)
Colon	1.73 (1.30, 2.16)	1.69 (1.23, 2.16)	1.91 (0.73, 3.10)
Lung	1.80 (1.20, 2.41)	1.73 (1.05, 2.41)	2.03 (0.70, 3.35)
Pancreas	1.96 (1.28, 2.63)	2.00 (1.27, 2.72)	1.64 (0, 3.50)
Stomach	1.09 (0.71, 1.48)	1.17 (0.73, 1.60)	0.76 (0.02, 1.51)
Bladder	2.44 (1.06, 3.83)	2.74 (1.04, 4.44)	1.58 (0, 3.77)
Head and neck	2.65 (1.08, 4.21)	2.71 (0.94, 4.47)	2.41 (0, 5.75)
Kidney	1.78 (0.68, 2.89)	1.06 (0.13, 1.99)	5.54 (0.68, 10.39)
Biliary tract	1.03 (0.27, 1.80)	0.69 (0.01, 1.37)	3.00 (0, 6.39)
Brain and central nervous system	0.91 (0.11, 1.72)	1.01 (0.12, 1.89)	NC
Larynx	2.59 (1.00, 6.17)	5.02 (0, 11.98)	NC
Haematology malignancy	**5.87 (4.48, 7.27)**	**5.22 (3.80, 6.64)**	**9.89 (5.05, 14.74)**
Non-Hodgkin lymphoma	6.24 (4.20, 8.27)	5.33 (3.32, 7.34)	12.71 (4.41, 21.02)
Multiple myeloma	8.19 (3.90, 12.48)	8.95 (4.08, 13.82)	3.90 (0, 11.53)
Leukaemia	3.64 (1.58, 5.70)	2.87 (0.88, 4.86)	7.80 (0.16, 15.45)
Hodgkin lymphoma	7.53 (1.00, 16.05)	9.41 (0, 20.06)	NC

The standardised incidence ratio (SIR) was presented with 95% confidence interval. *CI*, confidence interval; *NC*, not calculated

## Discussion

In this retrospective population-based cohort study, we found that individuals with SLE were at increased risk of haematologic malignancy as well as solid malignancy in Korean patients compared to the general population. Female patients with SLE had a significantly increased risk of multiple myeloma, NHL and solid cancers such as breast or reproductive system, thyroid, liver, colon, lung, pancreas and bladder cancers, whereas male patients with SLE demonstrated an increased risk of developing NHL.

Our results are similar to the profile observed in other studies [12, 18]. A cohort study based on an international multi-centre showed an increased risk of NHL (SIR 4.39, 95% CI 3.46–5.49) and cancers of the vulva (SIR 3.78, 95% CI 1.52–7.78), lung (SIR 1.30, 95% CI 1.04–1.60), thyroid (SIR 1.76, 95% CI 1.13–2.61) and possibly liver (SIR 1.87, 95% CI 0.97–3.27) [11]. However, the risk of NHL, Hodgkin lymphoma and multiple myeloma with SIRs of 6.24 (95% CI 4.20–8.27), 7.53 (95% CI 1.00–16.05) and 8.19 (95% CI 3.90–12.48), respectively, were estimated to be higher in our study than those in the international cohort study. In addition, the risk of cancer of the breast or reproductive system was increased in our study, whereas these risks were decreased in the other study [12]. Some of these differences in outcomes may be associated with the difference in either population factor such as ethnicity or lifestyle and the variation in the study design.

A nationwide cohort study performed in Taiwan using national claims database had the study design was quite similar to our study and Asian ethnicity. This study demonstrated an increased risk of vulva/vagina (SIR 4.76, 95% CI 4.24–5.33), kidney (SIR 3.99, 95% CI 3.74–4.27), nasopharynx (SIR 4.18, 95% CI 3.93–4.45) and haematologic (SIR 4,96, 95% CI 4.79–5,14) malignancies in SLE patients [19]. These were similar to our results, but some differences in site-specific risk in malignancies are made from the enrolment period and the relatively small sample size of the previous study.

We also compared our results with previous studies performed in Korea. A previous study in Korea has also reported that female SLE patients have an increased risk of overall malignancy (SIR 1.45, 95% CI 0.74–.216) [14]. Inconsistent with our results, the published data described a decreased risk of thyroid cancer (SIR 0.98, 95% CI 0.00–3.85) in female SLE patients, whereas our analysis showed an increased risk (SIR 1.55, 95% CI 1.30–1.80). Otherwise, NHL was significantly increased by more than 15-fold (SIR 15.2, 95% CI 2.29–37.7), while our result shows 5.33 in SIR [14]. The reason for these differences is considered that the study collected from one single university hospital between 1997 and 2007. In addition, the sample size was less than 1000, which was too small to ensure the external validity of the study results.

Recently, another study based on the NHIS database in Korea was performed [13]. It reported that Korean patients with SLE had a higher risk of overall malignancy (odds ratio, OR, 1.44; 95% CI 1.327–1.559) and specific malignancies, such as cervical, thyroid, ovarian and oral cancer, as well as lymphoma, leukaemia and multiple myeloma, than controls. They used the same database for the selection of patients with SLE, but there were several differences compared to our study. We selected the NHIS-NSC database as a comparator and calculated the SIRs of each malignancy, whereas they used a retrospective cohort after 1:5 age and sex-matching. Hence, they could only present the increased risk with ORs. Definitions of inclusion and exclusion of SLE patients, wash-out period of malignancies and observational period of the study also differed from those of our study.

Despite some differences in the study design and data sources, the risk of overall malignancy in SLE patients has increased in all the studies. There could be various explanations for the aetiology and pathogenesis, though no clear mechanism has been established. First, immunosuppressive therapy could potentiate immune dysregulation and lead to developing cancer [20]. Some large-scale cohort studies have been conducted to identify the association between the development of malignancies and immunosuppressive agents [18, 21]. In a multi-centre cohort study, the cumulative dose of cyclophosphamide affected the risk of malignancy in SLE patients [22]. Second, genomic studies have identified mutational genes at increased risk of malignancy in SLE patients. Chronic inflammation probably contributes to oxidative damage, DNA bases breaks and DNA-protein crosslinks [23]. In addition, SLE is associated with polymorphisms in DNA repair genes [24]. The combination of chronic inflammation and genetic predisposition to impaired DNA repair may contribute to DNA damage and increased risk of malignancy in SLE patients [25]. Third, in response to chronic immune damage, the cytokines that are associated with cell survival and proliferation, such as B cell activating factor, are overexpressed. Dysregulated immune proliferation could promote the translocation and juxtaposition of an oncogene beside a gene regulating immune function. This is related to the development of haematologic malignancies, particularly NHL [26]. Finally, some studies have reported an increased risk of reproductive system cancer, particularly vulvar and cervical cancer. Regarding the increased risk, sex hormonal effects and the altered clearance of human papillomavirus (HPV) are important factors [27].

Although the risk of malignancy seems to be increased, there was not a positive association between expected mortality and SLE in cohort studies. A cohort study that included 9547 patients with SLE from 23 sites worldwide, reported no increase in the mortality for overall malignancy (SMR 0.8, 95% CI 0.6–1.0) [28]. A study of the mortality profile of SLE patients in France identified 1,593 deceased patients with SLE and reported an even lower SMR (0.40, 95% CI 0.34–0.48) for overall malignancy [29]. These results indicate that other causes, such as cardiovascular disease, infectious disease or socioeconomic status, could attribute to the premature death of SLE patients.

This study has several strengths. First, the subjects in our study consisted of all the SLE patients in Korea and the general population through the NHIS database which provides a large nationwide and highly representative sample. We reduced environmental effects and prevented a possible ascertainment bias. Second, the study population and follow-up were well defined because of the broad coverage of the Korean NHIS. Third, through the estimation of not only the crude IR but also the SIR, we could determine the increased risk of malignancies in SLE patients compared to the general population.

Our study has several limitations. First, patients who are diagnosed with SLE are followed up and undergo regular examinations more closely than the general population. This is a surveillance bias that could increase the incidence of malignancy. Second, although our study showed increased cancer risk of SLE patients with statistical significance, a relatively short observation period may be noted in this study. Also, we could not obtain statistical significance in the malignancy risk of male SLE patients due to the relatively small size of subjects calculated. It should be considered that the duration and size of the study need to be extended. Third, we could not analyse the risk factors for the malignancy in patients with SLE, since we used the prevalence cohorts in both SLE patients and the general population. Further research which determines whether the development of cancer in patients with SLE is associated with the disease itself or drugs including immunosuppressive agents recently approved. To identify the risk factors for the development of malignancies, an incident cohort study may be conducted to minimise the immortal time bias [30].

Through this study, we established the site-specific cancer risk of Korean patients with SLE from the perspectives of frequency and of increasing risk compared to the general population. Further studies are needed to detect risk factors for the development of each malignancy and their impact on the mortality of SLE patients in Korea.

## Conclusion

In conclusion, our study is a large-scale nationwide cohort study that supports evidence for increased risk of malignancy in SLE patients, not only in haematologic malignancies but also solid malignancies, compared to the general population.

## Data Availability

The datasets used and/or analysed during the current study are available from the corresponding author on reasonable request.

## Abbreviation

SLE: Systemic lupus erythematosus
NHL: Non-Hodgkin lymphoma
HIRA: Health Insurance Review and Assessment Service
NHIS-NSC: National Health Insurance Service-National Sample Cohort Database
RID: Rare intractable disease
ICD: International Classification of Diseases
IR: Incidence rate
PYs: Person-years
CI: Confidence interval
SIR: Standardised incidence ratio
